# Characterizing the small RNA transcriptome associated with COPD and ILD using next-generation sequencing

**DOI:** 10.1186/1753-6561-6-S6-P6

**Published:** 2012-10-01

**Authors:** Joshua D Campbell, Lingqi Luo, Gang Liu, Ji Xiao, Joseph Gerrein, Brenda J Guardela, John Tedrow, Yuriy O Aleksyev, Ivana V Yang, Mick Correll, Mark Geraci, John Quackenbush, Frank Sciurba, David A Schwartz, Naftali Kaminski, Marc E Lenburg, Jennifer Beane, Avrum Spira

**Affiliations:** 1 Bioinformatics Graduate Program, Boston University, Boston, MA, USA; 2 Section of Computational Biomedicine, Boston University, Boston, MA, USA; 3 Simmons Center for Interstitial Lung Disease and Department of Medicine, University of Pittsburgh Medical Center, Pittsburgh, PA, USA; 4 Department of Pathology and Laboratory Medicine, Boston University School of Medicine, Boston, MA, USA; 5 Center for Genes, Environment and Health and Department of Medicine, National Jewish Health, Denver, CO, USA; 6 Dana-Farber Cancer Institute and Harvard School of Public Health, Boston, MA, USA; 7 Department of Medicine, University of Colorado School of Medicine, Aurora, CO, USA

## Background

Despite the increasing public health burden associated with chronic obstructive pulmonary disease (COPD) and interstitial lung disease (ILD), the molecular mechanisms responsible for the pathogenesis of these diseases remain unclear. The goal of this study was to comprehensively profile the lung small RNA transcriptome via next-generation sequencing, and elucidate microRNAs that might contribute to COPD and ILD pathogenesis.

## Materials and methods

As part of the Lung Genomics Research Consortium, we sequenced the small RNA in lung tissue samples from subjects with COPD (*n* = 150) or ILD (*n* = 149), and from normal lung tissue (*n* = 65). 319 lung tissue samples were sequenced via multiplexing on the Illumina HiSeq 2000 (10 samples/lane) and 45 samples were sequenced on the Illumina GAIIx (one sample per lane). Reads were trimmed using the FASTX toolkit and aligned to hg19 using Bowtie. Reads mapping to a microRNA locus were grouped into those with the canonical seed and those with an alternative seed due to variation in the 5' start position (isomiRs). Negative binomial generalized linear models were used to identify microRNAs and isomiRs differentially expressed between phenotypes. The large RNA from a subset of these samples was also sequenced using the Illumina GAIIx (one sample per lane). A network was generated by identifying all microRNA-mRNA pairs that were significantly anti-correlated (Spearman, false discovery rate (FDR) <0.05) and had a predicted microRNA-binding site in the mRNA 3'UTR (TargetScan 6.0).

## Results

An average of 26.3 million and 7.1 million reads were sequenced per sample using the singleplex and multiplex protocols, respectively. An average of 73% of reads per sample aligned to the human genome with one or fewer mismatches at 10 or fewer locations. 287 novel microRNA precursors were predicted using the miRDeep algorithm. One of these candidates was validated by quantitative RT-PCR and found to be expressed across a range of human tissues. The expression of 309 canonical microRNAs was significantly different between patients with disease and controls (FDR <0.05). In addition, we found that 242 isomiRs from 159 different microRNA loci were also differentially expressed (FDR <0.05). We developed a network of microRNA-mRNA interactions by integrating small RNA and large RNA sequencing data generated on the same samples (*n* = 72). 2,133 genes in the network (65%) were predicted to be regulated by at least one differentially expressed isomiR. Both the canonical form and three isomiRs of miR-338-3p were significantly downregulated in ILD. Predicted targets of the canonical form of miR-338-3p were enriched in extracellular matrix genes while the predicted targets of a miR-338-3p isomiR were enriched in the Wnt signaling pathway (*P*<0.001), suggesting different roles for multiple forms of this microRNA in ILD.

## Conclusions

Our results demonstrate the power of deep sequencing to reveal additional complexity in the microRNA transcriptome, such as novel microRNAs and isomiRs. The disease-related patterns of microRNA expression can provide insights into the molecular pathogenesis of chronic lung diseases and novel targets for therapy.

